# Neuropsychiatric manifestations of COVID-19, potential neurotropic mechanisms, and therapeutic interventions

**DOI:** 10.1038/s41398-021-01629-8

**Published:** 2021-09-30

**Authors:** Ying Han, Kai Yuan, Zhe Wang, Wei-Jian Liu, Zheng-An Lu, Lin Liu, Le Shi, Wei Yan, Jun-Liang Yuan, Jia-Li Li, Jie Shi, Zhong-Chun Liu, Gao-Hua Wang, Thomas Kosten, Yan-Ping Bao, Lin Lu

**Affiliations:** 1 National Institute on Drug Dependence and Beijing Key Laboratory on Drug Dependence, Peking University, Beijing, China; 2 Peking University Sixth Hospital, Peking University Institute of Mental Health, NHC Key Laboratory of Mental Health (Peking University), National Clinical Research Center for Mental Disorders (Peking University Sixth Hospital), Peking University, Beijing, China; 3 School of Public Health, Peking University, Beijing, China; 4 Department of Psychiatry, Renmin Hospital of Wuhan University, Wuhan, China; 5 Division of Alcohol and Addiction Psychiatry, Baylor College of Medicine, Houston, TX USA; 6 Peking-Tsinghua Center for Life Sciences and PKU-IDG/McGovern Institute for Brain Research, Peking University, Beijing, China

**Keywords:** Psychiatric disorders, Pathogenesis

## Abstract

The coronavirus disease 2019 (COVID-19) pandemic has caused large-scale economic and social losses and worldwide deaths. Although most COVID-19 patients have initially complained of respiratory insufficiency, the presence of neuropsychiatric manifestations is also reported frequently, ranging from headache, hyposmia/anosmia, and neuromuscular dysfunction to stroke, seizure, encephalopathy, altered mental status, and psychiatric disorders, both in the acute phase and in the long term. These neuropsychiatric complications have emerged as a potential indicator of worsened clinical outcomes and poor prognosis, thus contributing to mortality in COVID-19 patients. Their etiology remains largely unclear and probably involves multiple neuroinvasive pathways. Here, we summarize recent animal and human studies for neurotrophic properties of severe acute respiratory syndrome coronavirus (SARS-CoV-2) and elucidate potential neuropathogenic mechanisms involved in the viral invasion of the central nervous system as a cause for brain damage and neurological impairments. We then discuss the potential therapeutic strategy for intervening and preventing neuropsychiatric complications associated with SARS-CoV-2 infection. Time-series monitoring of clinical–neurochemical–radiological progress of neuropsychiatric and neuroimmune complications need implementation in individuals exposed to SARS-CoV-2. The development of a screening, intervention, and therapeutic framework to prevent and reduce neuropsychiatric sequela is urgently needed and crucial for the short- and long-term recovery of COVID-19 patients.

## Introduction

Severe acute respiratory syndrome coronavirus (SARS-CoV-2) infection causes coronavirus disease 2019 (COVID-19). SARS-CoV-2 is a single-stranded positive-sense RNA virus, belongs to the beta coronavirus genus, and is genetically and structurally like SARS-CoV. Phylogenetic analysis of the complete viral genome revealed that SARS-CoV-2 was a close relative to SARS-CoV, with 89.1% nucleotide similarity and 79% genetic similarity [1, 2]. This virus has spread rapidly worldwide, having devastating consequences on healthcare systems, society, and economies.

Up to date, this virus has infected millions and affected billions of lives in more than 200 countries. The mortality rate varies dramatically from country to country, but deaths are age-dependent. Deaths of individuals 60 years of age and older account for more than 80% of all deaths in the United States and the United Kingdom [3, 4]. The elderly are more susceptible to COVID-19 and have a higher risk of morbidity and mortality than the general population. Many factors, including frailty, comorbidities (e.g., hypertension, diabetes, cardiovascular disease, and chronic respiratory disease), and compromised immune function may contribute to worse health outcomes and a high mortality rate. Cardiovascular disease, diabetes, hypertension, or other comorbidities, preexisting microvascular pathology may further facilitate the neuroinvasion of the coronavirus and contribute to the development of neuropsychiatric symptoms and neuropathology associated with the viral infection [5]. The presence of chronic neurological comorbidity is an independent predictor of all-cause mortality in hospitalized COVID-19 patients [6]. A cohort study of multiple sclerosis patients with COVID-19 identified age, neurological disability, and obesity as the independent risk factors associated with COVID-19 severity [7].

While commonly manifested as fever and cough, atypical neuropsychiatric symptoms are also frequently reported in COVID-19 patients and include delirium, confusion, and neurocognitive disorders. These neuropsychiatric symptoms further hinder and delay diagnosis and treatment and have short- and long-term impacts on population health [8]. Several studies report risk or prognostic factors that are associated with a fatal outcome in patients hospitalized with COVID-19 and include medical comorbidities, dyspnea, time from disease onset to hospitalization, high procalcitonin levels, and lymphocytopenia [9, 10]. Mechanistically, coronavirus can invade the central nervous system (CNS) through blood vessels and neuronal retrograde pathways, thereby causing brain injury and dysfunction of the cardiorespiratory center in the brainstem, manifested as neurological symptoms and respiratory failure in infected animals and patients [11–13]. Conditional bursting pacemaker neurons in the brainstem region are crucial for the generation of respiratory rhythm [14], and SARS-CoV-2 may affect these respiratory control mechanisms, leading to indirect respiratory dysfunction in addition to primary pulmonary injury [15].

Historically, two similar human coronaviruses, SARS-CoV and the Middle East respiratory syndrome (MERS)-CoV, have caused severe acute respiratory syndrome outbreaks in 2003 and 2012. During these two pandemics, neuropsychiatric complications included narcolepsy, seizures, encephalitis, encephalopathy, Guillain–Barrè syndrome (GBS), and neuromuscular and psychiatric disorders [16–19]. These neuropsychiatric sequelae were closely associated with morbidity and risk of mortality. Emerging evidence suggests that SARS-CoV-2 presents an analogous neurotropic property and its infection can result in acute and long-term neuropsychiatric consequences. Neurotropic RNA coronaviruses could disrupt the blood–brain barrier (BBB), invade the CNS, and affect neuroimmune interactions through macrophages, microglia, or astrocytes [20, 21]. Clinical trials reported that patients with COVID-19-related pneumonia exhibited neurological disorders (e.g., stroke, encephalopathy, encephalitis, delirium, and GBS) and psychiatric disorders (e.g., depression, anxiety, insomnia, and post-traumatic stress disorder) [22–25]. Moreover, in some cases, neurological or psychiatric complications may precede or present without typical respiratory manifestations [26]. A recent meta-analysis reported that psychiatric and neurological disorders increased the susceptibility to COVID-19, illness severity, and mortality [27].

Here, we provide a comprehensive overview of the prevalence and presentation of neuropsychiatric manifestations of COVID-19 in patients based on epidemiological studies, clinical reports, and neuroimaging findings and discuss potential neuroinvasive mechanisms and pathways that contribute to these neuropathological changes and CNS dysfunction after SARS-CoV-2 infection. We also discuss possible and promising interventions that prevent and reduce these neuropsychiatric complications.

## The presence and prevalence of neuropsychiatric manifestations in COVID-19 patients

Neurological and psychiatric complications of COVID-19 are increasingly reported, but most are individual cases or case series. Headache, anosmia, and myalgia are most commonly reported in patients infected with SARS-CoV-2. SARS-CoV-2 infection can attack the CNS and induce spine demyelinating lesions [28], which could further lead to neuropsychiatric symptoms affecting cognitive, affective, behavioral, and perceptual domains. These neuropsychiatric symptoms, including cerebrovascular, psychiatric, and neuromuscular disorders, frequently occur in elderly patients and individuals with multiple comorbidities or severe infection. Both SARS and MERS are associated with delirium, depression, anxiety, memory impairment, and insomnia during the acute phase. Depression, insomnia, anxiety, memory impairment, and sleep disorders are frequently reported during the post-illness phase [23]. A significant proportion of patients with COVID-19 develop delirium, agitation, altered consciousness, and other neuropsychiatric symptoms, including encephalopathy, encephalitis, depression, anxiety, and post-traumatic stress disorder [23].

A UK-wide surveillance study of acute neurological and psychiatric complications in 153 COVID-19 patients demonstrated that cerebrovascular events (62%) and altered mental status (31%, including encephalopathy, encephalitis, and psychiatric disorders, were reported, often occurring in younger patients [29]. An observational series of 58 COVID-19 patients in Strasbourg, France reported encephalopathy, prominent agitation and confusion, corticospinal tract signs, and acute ischemic strokes [30]. A tertiary-care hospital at Karachi, Pakistan reported on 350 patients with COVID-19 describing headache (6%), vertigo (3.4%), numbness/paresthesia (3.1%), impaired consciousness (2%), hyposmia/anosmia (1.4%), and encephalitis (0.9%) [31]. A retrospective, observational case series of 214 patients in Wuhan, China found that 78 patients (36.4%) had neurologic manifestations, including acute cerebrovascular diseases, impaired consciousness, and skeletal muscle injury [32]. Analysis of data from 86 critically ill COVID-19 patients at the intensive care unit (ICU) of Tongji Hospital, Wuhan, China showed that 26 patients (30.2%) presented with neurological symptoms including delirium, stroke, cerebrovascular, and neuromuscular diseases [33]. A retrospective multicenter cohort study of 917 patients in three regions in China demonstrated that new-onset critical neurologic events, mainly impaired consciousness and stroke, occurred in 3.5% of the total population and in 9.4% of severe or critical patients [34]. A prospective multicenter observational study in New York City showed that 13.5% (606/4491) hospitalized COVID-19 patients developed a new neurological disorder including encephalopathy (309/606, 51%), strokes (84/606, 14%), seizures (74/606, 12%), and hypoxic/ischemic brain injury (65/606, 11%), and these disorders led to higher rates of in-hospital mortality and lower rates of discharge home [35]. A survey of physician-reported neurological symptoms in COVID-19 patients from Italy showed that 87.3% of practitioners reported neurological symptoms, mainly mild and nonspecific manifestations such as headache, myalgia, and loss of smell [36].

GBS and myelitis are also reported in COVID-19 patients, indicating a post-infective autoimmune reaction in peripheral nerves [37]. COVID-19 patients frequently presented with stroke and subsequent mortality when complicated by older age, comorbidities, and severe respiratory symptoms [38]. A meta-analysis of 58,104 COVID-19 patients revealed a 0.46% hemorrhagic stroke rate and a 1.11% ischemic stroke rate, with mortality rates of 44.7% for hemorrhagic and 36.2% for ischemic stroke [39]. Evidence from tissue histology, neuroimaging, and clinic symptoms revealed that 1.4% of COVID-19 patients (23/1683) developed cerebral ischemia, intracerebral hemorrhage, or encephalopathy [40]. A possible pathophysiology for this cerebrovascular damage may be BBB dysfunction and subsequent cytokine release induced by SARS-CoV-2 infection of the brain itself [41].

During the COVID-19 pandemic, psychiatric disorders such as depression, anxiety, post-traumatic stress disorder, and insomnia have also been frequently reported in COVID-19 patients, vulnerable populations, healthcare workers, and even the general population [23, 42–48]. Poor sleep is associated with worsened clinical outcomes in hospitalized patients with COVID-19, and long-term sustainable improvements in sleep quality are needed for this subpopulation [49, 50]. The prevalence and severity of these psychiatric symptoms, attention deficits, and hyperactivity symptoms increased the risk for problematic internet use during the COVID-19 pandemic [51]. Moreover, a case series reported that critically ill COVID-19 patients with multiple acute bilateral ischemic lesions exhibited alterations of mental status but no neurological deficits [52]. A group of experts convened by the UK Academy of Medical Sciences and the mental health research charity also advocate monitoring and evaluating brain function, and mental health issues such as anxiety, depression, insomnia, self-harm, and suicide in COVID-19 patients and related vulnerable populations [53]. A Nationwide Cohort Study also revealed that hospitalization with infection increased the risk of death by suicides in prospective and dose–response relationships [54]. Another cohort study demonstrated that a history of schizophrenia spectrum disorder was significantly associated with an increased risk for mortality among COVID-19 patients [55]. However, it is difficult to distinguish whether this high prevalence is due to direct SARS-CoV-2 infection or the adverse psychological effects of other social and environmental factors such as social distancing and quarantine, self-isolation, changes in sleep and lifestyle behaviors, fear of death, and economic burden [56].

Overall, neurologic and neuropsychiatric manifestations, such as alterations of mental status and stroke, are common among hospitalized COVID-19 patients and could be predictors of disease severity and mortality [57, 58]. A retrospective cohort study of 236,379 COVID-19 survivors revealed that the incidence of neurological or psychiatric morbidity (e.g., intracranial hemorrhage, ischemic stroke, dementia, and anxiety disorder) was 33.6% in the 6 months follow-up, indicating the neuropsychiatric sequela was long-lasting [59]. An international cohort study found that neurological symptoms did not recover in COVID-19 patients at 7-month follow-up [60]. Clinicians should seriously consider these neurological and neuropsychiatric symptoms to avoid delayed diagnosis or misdiagnosis and to reduce the risk of death.

## Neuroimaging and neurochemical findings of brain dysfunction in COVID-19 patients

Early brain imaging and neurochemical examinations in COVID-19 patients with neuropsychiatric symptoms are critical and important for timely interventions that can improve clinical outcomes. Moreover, advanced neuroimaging and evaluation of biomarkers are critical means in the clinical evaluation and diagnosis of brain injury and neurological disorders. We summarize recent findings on neuroimaging, electroencephalography (EEG), and neurochemical biomarkers that reflect CNS dysfunction in COVID-19 patients, especially those with neuropsychiatric manifestations (Table 1).

**Table 1:** Neuroimaging and neurochemical findings of brain dysfunction in COVID-19 patients, especially those with neuropsychiatric manifestations.

Study	Region	Participants	Sample size	Study design	Symptoms	Detection method	Main findings
Neuroimaging							
Coolen et al., 2020 [68]	Belgium	COVID-19 decedents	19	Case series	Headache, agitation, confusion, disorientation, seizures	MRI	Subcortical microbleeds and macrobleeds, cortico/subcortical edematous changes, deep white matter changes
Delorme et al., 2020 [64]	France	COVID-19-related encephalopathy	4	Case series	Various degrees of cognitive impairment with predominantly frontal lobe impairment	MRI, FDG-PET/CT	Frontal hypometabolism, cerebellar hypermetabolism
Freeman et al., 2020 [62]	USA	Inpatients with COVID-19	59	Case series	Not specified	MRI	Six (10.2%) had MRI findings suspicious of COVID-19-related disseminated leukoencephalopathy
Jensen et al., 2021 [70]	UK	Patients with fatal COVID-19	2	Case series	Not specified	Coronial autopsy	Cerebral cortical infarction, brainstem encephalitis
Kremer et al., 2020 [66]	France	Severe COVID-19 with neurologic manifestations	37	Cohort	Alterations of consciousness (27), pathological wakefulness after sedation (15), confusion (12), agitation (7)	MRI	Signal abnormalities in the medial temporal lobe, non-confluent multifocal white matter hyperintense lesions on FLAIR and diffusion with variable enhancement, associated with hemorrhagic lesions and extensive and isolated white matter microhemorrhage
Lu et al., 2020 [65]	China	COVID-19 patients	60	Prospective cohort	Mood changes (25), fatigue (16), headache (15), vision changes (13), myalgia (9), mobility impairment (7), memory loss (8), taste loss (4), limb numbness (4), tremor (4). loss of smell (2), hearing loss (1)	MRI	Recovered COVID-19 patients were more likely to have enlarged olfactory cortices, hippocampi, insulas, Heschl’s gyrus, Rolandic operculum, and cingulate gyrus and a general decline of mean diffusivity, axial diffusivity, and radial diffusivity, accompanied by an increase in white matter fractional anisotropy
Paterson et al., 2020 [188]	UK	COVID-19 patients	43	Case series	Encephalopathy, neuroinflammatory syndrome, stroke, transverse myelitis	MRI, CSF	SARS-CoV-2 infection was associated with a wide spectrum of neurological syndromes that affect the whole neuraxis
Poyiadji et al., 2020 [67]	USA	COVID-19-associated encephalopathy	1	Case report	Cough, fever, altered mental status	CT, MRI	Hemorrhagic rim enhancing lesions within bilateral thalami, medial temporal lobes, and subinsular regions
Sharifi-Razavi et al., 2020 [63]	Iran	Case of 79-year-old man	1	Case report	Fever and cough, acute loss of consciousness, rapid heart rate, rapid breathing	CT	Massive intracerebral hemorrhage in the right hemisphere, accompanied by intraventricular and subarachnoid hemorrhage
Solomon et al., 2020 [69]	USA	COVID-19 patients who died in hospital	18	Case series	Myalgia (3), headache (2), decreased taste (1)	CT, autopsy	Acute hypoxic injury in cerebrum and cerebellum in all patients, with loss of neurons in the cerebral cortex, hippocampus, and cerebellar Purkinje cell layer but no thrombi or vasculitis
Zanin et al., 2020 [28]	Italy	COVID-19 patient	1	Case report	Seizures, anosmia, ageusia	MRI	Brain MRI revealed alterations of periventricular white matter, with normal chemical and physical CSF examination
EEG							
Canham et al., 2020 [73]	UK	Cases of COVID-19 admitted to intensive care unit	10	Case series	Delirium with visual hallucinosis and drowsiness, unresponsive, drowsy	EEG	EEG features in severe COVID-19 indicated generalized symmetrical slowing
Chen et al., 2020 [74]	USA	Critically ill patients	5	Case series	All patients had encephalopathy and three also had seizure-like movements	EEG	EEG showed nonspecific markers of encephalopathy, including diffuse slowing and generalized rhythmic delta activity
De Stefano et al., 2020 [76]	Switzerland	COVID-19 patient undergoing mechanical ventilation	1	Case report	Altered mental status	EEG	Cerebral microbleeds, focal dysfunction
Louis et al., 2020 [75]	USA	COVID-19 patients	22	Retrospective cohort	Intracerebral hemorrhage (1), acute ischemic stroke (1), imaging concerning for possible ischemia (2)	EEG	Various epileptiform abnormalities on EEG, a higher proportion of patients with electrographic seizures
Vespignani et al., 2020 [72]	France	Severe COVID-19-infected patients	5	Case series	Face and eye myoclonus, delayed awakening, poor arousal, confusion, lethargy	EEG	High-amplitude frontal monomorphic delta waves without epileptic activity, indicating potential CNS injury
Neurochemical biomarkers							
Alexopoulos et al., 2020 [85]	Greece	COVID-19 patients with signs of encephalopathy	8	Case series	Pulmonary imaging of diffuse infiltrates and ground-glass opacities, with renal failure in four patients, hepatic failure in one patient, and agitated confusion in one patient	Serum, CSF	High-titer anti-SARS-CoV-2 antibodies in both serum and CSF, with 14-3-3-protein positivity in CSF in four patients
Ameres et al., 2020 [78]	Germany	Healthcare workers (28 positive for COVID-19, 72 negative for COVID-19)	100	Prospective cohort	Mild-to-moderate symptoms, no or only minor neurological symptoms, including anosmia and headache	Serum	Mild-to-moderate COVID-19 was associated with higher serum NfL levels
Domingues et al., 2020 [87]	Brazil	42-year-old patient	1	Case report	Mild respiratory symptoms, neurological manifestations	CSF	SARS-CoV-2 RNA detected in CSF
Edén et al., 2021 [82]	Sweden	COVID-19 patients with neurologic abnormalities	6	Case series	All had respiratory symptoms with hypoxemia and encephalopathy	CSF	Marked elevations of two soluble inflammatory biomarkers in all patients, abnormal CSF NfL in two patients
Espíndola et al., 2020 [90]	Brazil	Patients with COVID-19	8	Case series	Meningoencephalitis (1), encephalitis (1), facial palsy (2), delirium (2), intracranial hypertension (1), new daily persistent headache (1)	CSF	Undetectable or extremely low levels of SARS-CoV-2 RNA in CSF
Helms et al., 2020 [30]	France	Patients with COVID-19 and ARDS	58	Case series	Agitation (40), corticospinal tract signs (39), dysexecutive syndrome (14)	CSF, MRI	Negative RT-PCR for SARS-CoV-2 in CSF
Kanberg et al., 2020 [77]	Sweden	Patients with confirmed COVID-19	47	Cross-sectional	Symptoms of confusion (4), single seizure episode (1)	Plasma	Higher plasma concentrations of NfL and glial fibrillary acidic protein in severe COVID-19 patients
Kanberg et al., 2021 [80]	Sweden	Patients with confirmed COVID-19	100	Cohort	Headache (41%) and dysgeusia (11%), myalgia, hyposmia, dysgeusia, altered cognition	Plasma	Post-COVID-19 neurological sequelae not accompanied by ongoing CNS injury
Moriguchi et al., 2020 [86]	Japan	Meningitis associated with SARS-CoV-2	1	Case report	Convulsions accompanied by unconsciousness, fatigue, fever	CSF, serum	specific SARS-CoV-2 RNA detected in CSF
Oussalah et al., 2020 [81]	France	Patients with severe COVID-19	162	Longitudinal cohort	Not specified	Blood, urine	C-reactive protein and urea nitrogen associated with risk of COVID-19-related acute respiratory failure and death
Paterson et al., 2021 [83]	USA	COVID-19 patients	152	Prospective cohort	Not specified	Serum, CSF	High concentrations of NfL in CSF among patients with CNS inflammation
Prudencio et al., 2021 [79]	USA	Patients hospitalized with COVID-19	142	Cohort	Not specified	Serum	Higher NfL in serum in patients with COVID-19 compared with healthy controls, higher serum NfL levels associated with worse clinical outcomes
Ye et al., 2020 [88]	China	COVID-19 case presented as encephalitis	1	Case report	Fever, shortness of breath, myalgia, confusion, meningeal irritation signs, extensor plantar response	CSF	Negative test for SARS-CoV-2 in CSF

*MRI* magnetic resonance imaging, *FDG-PET/CT* [^18^F]fluoro-2-deoxy-d-glucose-positron emission tomography/computed tomography, *CSF* cerebrospinal fluid, *CT* computed tomography, *EEG* electroencephalogram, *CNS*, central nervous system, *NfL* neurofilament light-chain protein.

### Diagnostic MRI/PET/CT findings

Many severe COVID-19 patients who are admitted to an ICU risk infection spread when transported to imaging suites. In response to this risk, clinicians should consider a recently developed novel portable, low-field magnetic resonance imaging (MRI) device to evaluate neurological injury such as stroke and hemorrhage at the bedside of critically ill ICU patients [61]. Brain MRI examinations revealed uncommon but important findings of disseminated leukoencephalopathy in COVID-19 patients with neurologic symptoms [62]. Brain computed tomography (CT) scan in a patient with SARS-CoV-2 infection revealed a massive intracerebral hemorrhage in the right hemisphere, accompanied by intraventricular and subarachnoid hemorrhage [63]. Brain [^18^F]fluoro-2-deoxy-d-glucose-positron emission tomography/CT imaging in four cases of COVID-19-related encephalopathy showed consistent frontal hypometabolism and cerebellar hypermetabolism [64]. An MRI-based 3-month follow-up study showed that 55% of COVID-19 patients who presented with neurological symptoms had microstructural and functional brain integrity disruption during this recovery stage [65]. Besides ischemic infarction, signal abnormalities in the medial temporal lobe, non-confluent multifocal white matter hyperintense lesions, and extensive and isolated white matter microhemorrhages are frequently found in severe COVID-19 patients with neurological symptoms [66]. A COVID-19 patient presenting with altered mental status showed acute necrotizing encephalopathy on CT and MRI [67].

Early postmortem brain MRI in COVID-19 non-survivors have demonstrated white matter changes, brain hemorrhages, and encephalopathy without brainstem changes, which all may result from BBB impairment [68]. Neuropathology in 18 COVID-19 non-survivors uncovered acute hypoxic injury in the cerebrum and cerebellum, with neuronal loss in the cerebral cortex, hippocampus, and cerebellar Purkinje cell layer, but no encephalitis [69]. Coronial autopsies of two fatal COVID-19 patients showed cerebral cortical infarction and brainstem encephalitis, but found no SARS-CoV-2 RNA in these postmortem brain tissues using RNAscope in situ hybridization and reverse transcription-polymerase chain reaction (RT-PCR) [70]. These neuropathological findings may be related to hyperinflammatory and hypercoagulable status induced by SARS-CoV-2 infection.

### Diagnostic EEG findings

EEG has shown no reliable findings related to COVID-19 [71]. EEG examinations in five severe COVID-19 patients showed high-amplitude frontal monomorphic delta waves without epileptic activity, indicating potential CNS injury [72]. Two case series reported generalized symmetrical slowing as predominant EEG features of encephalopathy in patients with severe COVID-19, and EEG monitoring is very important in this population to identify status epilepticus and thus guide timely anti-seizure interventions [73, 74]. Continuous EEG was also recommended to discover asymptomatic seizures or to identify status epilepticus in COVID-19 patients [75]. EEG-MRI examinations have revealed cerebral microbleeds and focal dysfunction in critical illness COVID-19 patients with acute neurological complications after ruling out nonconvulsive status epilepticus [76].

### Diagnostic neurochemical findings

Two plasma biomarkers of CNS injury, neurofilament light-chain protein (NfL, a marker of neuroaxonal injury) and glial fibrillary acidic protein (GFAP, a marker of astrocytic activation/injury), were increased in severe COVID-19 patients [77]. Another study in healthcare workers demonstrated that mild-to-moderate COVID-19 was associated with increased serum NfL levels, indicating the potential neurodestructive capability of SARS-CoV-2 [78]. Higher serum NfL concentrations were associated with worse clinical outcomes, such as mechanical ventilation and ICU admission, in hospitalized COVID-19 patients [79]. A longitudinal study showed that elevated NfL and GFAP concentrations normalized at 6-month follow-up, regardless of prior disease severity or persisting neurological symptoms, in all COVID-19 patients [80]. A longitudinal cohort study with time-series design to estimate the spectrum of biochemical dataset suggests two other biochemical markers, C-reactive protein (CRP) and urea nitrogen, are associated with the risk of COVID-19-related acute respiratory failure and death [81]. A case series showed that levels of biomarkers of inflammation in cerebrospinal fluid (CSF), including neopterin and β2-microglobulin, increased in six COVID-19 patients with neurological symptoms, including encephalopathies, suspected meningitis, and dysgeusia [82]. A large prospective biomarker study showed that serum NfL levels increased across hospitalized COVID-19 patients despite neurological manifestations, whereas CSF NfL levels increased specifically in patients with CNS inflammation, including encephalitis and acute disseminated encephalomyelitis, but not in patients with encephalopathy or GBS [83]. Moreover, a previous study evaluated nine antiphospholipid antibodies and found that anti-phosphatidylserine/prothrombin IgG was associated with COVID-associated neurological manifestations, specifically acute disseminated encephalomyelitis [84]. This neurochemical evidence supports the presence of neuroinflammation and microvascular and CNS injury in the acute phase of the disease.

Enzyme-linked immunosorbent assay (ELISA) revealed that high-titer anti-SARS-CoV-2 antibodies are detectable in both serum and CSF of patients with encephalopathy and comatose. These patients showed a breakdown of BBB integrity and neurodegeneration as indicated by finding albumin and 14-3-3-protein in the CSF [85]. In addition, SARS-CoV-2 RNA detection in the CSF could provide direct evidence to support the neurotropism theory. Recently, the first case of positive SARS-CoV-2 RT-PCR results in the CSF of COVID-19 patients with meningitis/encephalitis was reported [86], supporting neuroinvasion of the CNS after SARS-CoV-2 infection. SARS‑COV‑2 RNA was also detected in the CSF sampling of a patient with neurological manifestations of demyelinating disease, although the respiratory symptoms were mild [87]. Other studies using RT-PCR assays of CSF samples from COVID-19 patients with neurological damage have been negative for SARS-CoV-2 [28, 30, 76, 88], which may be due to low sensitivity or diagnostic complications of the method (i.e., false-negative results), CSF clearance, low CSF virus titer, and delayed sampling [89, 90].

Overall, the neuroimaging (MRI/CT/PET) and neurochemical (RT-PCR assays/ELISA) methods could help to identify candidate biomarkers to access the effects of SARS-CoV-2 infection on the CNS function and brain inflammation status [53]. Combined with clinical symptoms, self-reporting, and behavioral testing of emotional and cognitive domains, comprehensive diagnosis of disease and implicating proper interventions could be achieved.

## The potential mechanisms underlying the invasion of SARS-CoV-2 into the nervous system

**Fig. 1:**
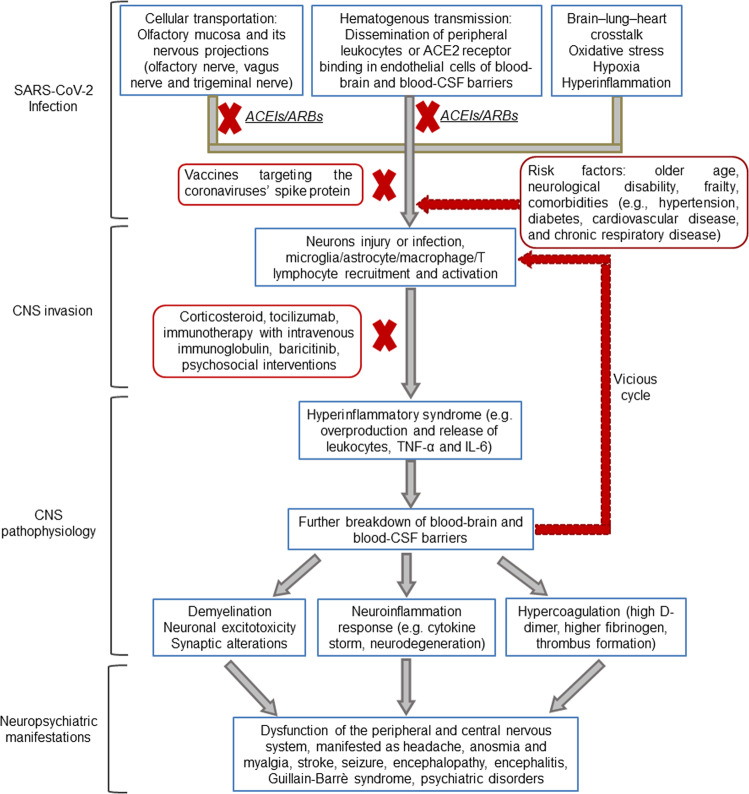
Neuropsychiatric manifestations, possible mechanisms of neurological impairments after SARS-CoV-2 infection, and potential therapeutic interventions.

Growing and convincing evidence supports the neurotropism of SARS-CoV-2 (Table 2), similar to other coronaviruses. Quantitative data for tropism, replication kinetics, and cell damage revealed that SARS-CoV-2 modestly replicated in neuronal cells, highlighting the potential that this virus can cause neuropsychiatric manifestations in COVID-19 patients [91]. This virus could affect the CNS and cause brain damage and neuropsychiatric alterations through several pathways (Fig. 1).

**Table 2:** Evidence supporting the direct neurotropic effects of SARS-CoV-2.

Models	Targets	Main findings	Methods	Immunostaining (colocalization with SARS-CoV-2 spike protein or nucleoprotein)	References
hPSC-derived neural progenitor cells, neurospheres, and brain organoids	Cortical neurons and neural progenitor cells	Human neural progenitor cells and neurospheres are permissive to SARS-CoV-2 infection and supported productive virus replication; SARS-CoV-2 can directly infect cortical neurons and neural progenitor cells in brain organoids	qRT-PCR; electron microscopy; immunofluorescence	Neuronal cell marker TUJ1, and proliferation neural progenitor cell marker NESTIN	Zhang et al., 2020 [96]
hPSC-derived monolayer brain cells, region-specific brain organoids, and choroid plexus organoids	Neurons, astrocytes, choroid plexus epithelial cells; cortical, hippocampal, hypothalamic, and midbrain regions	SARS-CoV-2 sparsely infects human neurons and astrocytes, but robustly infects choroid plexus epithelial cells; SARS-CoV-2 increases cell death in choroid plexus organoids	qRT-PCR; immunofluorescence; single-cell RNA-seq	Neuronal marker doublecortin, astrocyte marker GFAP, choroid plexus marker transthyretin	Jacob et al., 2020 [99]
hPSC-derived brain organoids	Choroid plexus epithelial cells	SARS-CoV-2 spike pseudovirus and live virus infects choroid plexus epithelial cells, but not neurons or glia; SARS-CoV-2 damages the choroid plexus epithelium and disrupts the blood–CSF barrier	qRT-PCR; immunofluorescence; single-cell RNA-seq	Choroid plexus marker transthyretin	Pellegrini et al., 2020 [101]
hPSC-derived neuroprogenitor cells and BrainSphere	Neuronal cell body and neurite structures	SARS-CoV-2 infects neuronal cell body and neurite structures with the replication of the virus	qRT-PCR; immunofluorescence	Neuronal marker MAP2	Bullen et al., 2020 [105]
hPSC-derived brain organoids; mice overexpressing human ACE2; brain autopsy samples of deceased COVID-19 patients	Neurons; forebrain cortex, cerebellum, dentate gyrus, the globus pallidus	SARS-CoV-2 infects human brain organoids accompanying metabolic changes and induces cell death; SARS-CoV-2 neuroinvasion in vivo in mice; the presence of SARS-CoV-2 in cortical neurons of autopsy samples	qRT-PCR; electron microscopy; immunofluorescence; single-cell RNA-seq	Neuronal marker MAP2, vascular endothelium marker CD31, and podocalyxin	Song et al., 2021 [106]
hPSC-derived cortical neurons and brain organoids	Cortical neuron	SARS-CoV-2 preferably targets cortical neurons of brain organoids and causes aberrant Tau localization and neuronal death	qRT-PCR; immunofluorescence; ELISA	Neuronal cell marker TUJ1	Ramani et al., 2020 [108]
hPSC-derived cells and organoids	Dopaminergic neurons	Dopaminergic neurons, but not cortical neurons or microglia, were permissive to SARS-CoV-2 infection	qRT-PCR; immunofluorescence; single-cell RNA-seq	Dopaminergic neuronal marker FOXA2 and MAP2, cortical neuronal marker beta III-tubulin, microglial marker Iba1	Yang et al., 2020 [110]
Human embryonic stem cell-derived monolayer cortical neurons and dorsal forebrain organoids	Cortical neuron	SARS-CoV-2 pseudovirus infects only 10% of cortical neurons	Immunofluorescence	Neuronal cell marker TUJ1	Yi et al., 2020 [107]
hPSC-derived neurons, astrocytes, and brain organoids	Neurons and astrocytes	SARS-CoV-2 infects hiPSC-derived neurons, astrocytes, and brain organoids; an increased rate of SARS-CoV-2 infection in ApoE4 neurons and astrocytes	qRT-PCR; immunofluorescence	Neuronal cell marker TUJ1 and MAP2; astrocyte marker SOX9 and GFAP	Wang et al., 2021 [97]
hPSC-derived neural progenitor cells, neurons, astrocytes, and cerebral organoids	Neurons and astrocytes	SARS-CoV-2 infects human cerebral organoids; SARS-CoV-2 infects neurons more efficiently than neural progenitor cells and astrocytes	qRT-PCR; immunofluorescence	Neuronal marker MAP2 and β-tubulin-III; astrocyte marker GFAP	Tiwari et al., 2021 [109]
hPSC-derived cortical organoids	Astrocytes	SARS-CoV-2 infects astrocytes in both primary human cortical tissue and human stem cell-derived cortical organoids	Immunofluorescence	Astrocyte marker GFAP; neuronal marker NeuN	Andrews et al., 2021 [111]
hPSC-derived cortical organoid and pericyte containing cortical organoids	Pericyte-like cells and astrocytes	SARS-CoV-2 infects pericyte-like cells and astrocytes	qRT-PCR, immunofluorescence, single-cell RNA-seq	Pericyte markers (NG2 and PDGFR-β)	Wang et al., 2021 [102]
hPSC-derived neural cultures	Neurons	SARS-CoV-2 does not replicate and only infects a minority of individual mature neurons but induces the production of type III interferons and interleukin-8	Immunofluorescence	Neuronal marker (MAP2), astrocyte marker (GFAP), neural progenitor cell marker (SOX2)	Bauer et al., 2021 [103]
Human embryonic stem cell-derived cortical organoids	Neurons, astrocytes, choroid plexus cells	SARS-CoV-2 only minimally infected neurons, but preferentially infected astrocytes and choroid plexus cells	Immunofluorescence	Neuronal markers (DCX and MAP2), choroid plexus marker (5-HT_2C_), astrocytic marker (GFAP and ALDH1L1)	McMahon et al., 2021 [100]
Deer mouse model of SARS-CoV-2 infection	Neurons and microglia	The presence of SARS-CoV-2 in neurons and microglia of the afferent nerves (trigeminal nerve) and in the glomerular layer of the olfactory bulb	Immunofluorescence; ELISA	Neuronal marker MAP2, microglial marker Iba1	Fagre et al., 2021 [131]
Rhesus monkey model of SARS-CoV-2 infection	Neurons, astrocytes, microglia, olfactory bulb, olfactory trigone, entorhinal area, hippocampus, thalamus, medulla oblongata, frontal lobe, occipital lobe	Intracranial inoculation with SARS-CoV-2 caused the distribution of viral antigen nucleoprotein in neurons, astrocytes, and microglial cells in multiple brain regions	qRT-PCR, immunofluorescence, single-cell RNA-seq	Neuronal marker (NeuN), astrocytic marker (GFAP_, microglial marker (Iba1)	Jiao et al., 2021 [132]
Brain autopsy samples of deceased COVID-19 patients	Neurons and cerebral vascular endothelial cells; medulla oblongata and cerebellum	the presence of SARS-CoV-2 RNA and protein in medulla oblongata and cerebellum; the presence of SARS-CoV-2 protein in the olfactory mucosa and its nervous tissues; the presence of SARS-CoV S protein in the endothelial cells of small brain vessels	qRT-PCR; electron microscopy; immunofluorescence	Neuronal marker TUJ1, NF200, and OMP	Meinhardt et al., 2020 [133]
Brain autopsy samples of deceased COVID-19 patients	Medulla oblongata and cranial nerves	The presence of SARS-CoV-2 viral proteins in cranial nerves originating from the lower brainstem and in isolated cells of the medulla oblongata	qRT-PCR; immunohistochemistry	—	Matschke et al., 2020 [134]

*hPSC* human pluripotent stem cells, *CSF* cerebrospinal fluid, *TuJ1* class III β-tubulin, *GFAP* glial fibrillary acidic protein, *MAP2* microtubule-associated protein 2, *Iba1* ionized calcium-binding adaptor molecule 1, *NF200* neurofilament 200, *OMP* olfactory membrane protein.

### Neurotropism of SARS-CoV-2 using human pluripotent stem cell technology and brain organoids

Human pluripotent stem cell (hPSC) technology has been successfully used to study viral infections of the CNS such as the Zika virus, and have immensely implicated to identify the specificity of infected cell types and brain organoids, model neurodevelopmental or neurodegenerative disorders, elucidate disease progress and mechanisms, and develop potential therapeutic agents [92–94]. Using these models including human neural progenitor cells, neurospheres, monolayer brain cells, and three-dimensional (3D) region-specific brain organoids, the emerging experimental evidence has demonstrated that SARS-CoV-2 can invade the human CNS and infect various cell types (Table 2) [95–98].

SARS-CoV-2 was found to sparsely infect human neurons, but robustly infect choroid plexus epithelial cells and increase cell death, indicating that SARS-CoV-2 may invade the CNS by acting on the blood–CSF barrier in the choroid plexus [99, 100]. SARS-CoV-2 spike pseudovirus and live virus specifically infected choroid plexus epithelial cells, but not neurons or glia, and disrupted the blood–CSF barrier function in human iPSC-derived brain organoids [101]. By integrating pericyte-like cells into hPSC-derived cortical organoids, Wang et al. found that pericyte-like cells were extensively infected by SARS-CoV-2 and facilitated viral spread to astrocytes [102]. Several studies showed that SARS-CoV-2 does not replicate in hiPSC-derived neural cultures, arguing that most brain damage that is caused by SARS-CoV-2 infection is attributable to local immune responses rather than robust viral replication in the CNS [103, 104]. However, other studies using human brain organoids found the replication of SARS-CoV-2 in the neuronal cell body and neurite structures, accompanying metabolic changes [105–107]. Furthermore, SARS-CoV-2 preferably targets cortical neurons of 3D human brain organoids, where it has altered Tau distribution, and phosphorylation, which increased expression of neurodegeneration genes and induced neuronal death, providing further insights into the neuropathogenesis of SARS-CoV-2 infection [108, 109]. Yang et al. found that the SARS-CoV-2 pseudo-entry virus and SARS-CoV-2 virus infects dopaminergic neurons, but not cortical neurons or microglia [110]. Andrews et al. found that SARS-CoV-2 infects astrocytes in both primary human cortical tissue and human stem cell-derived cortical organoids [111]. Although 2D or 3D in vitro experimental models could not perfectly recapitulate the complex clinical symptoms and multifactorial cellular effects in COVID-19 patients, the development of mature and complex brain organoids comprising neurons, astrocytes, microglia, vasculature, and choroid plexus provides promising means to explore the neuropathogenesis of SARS-CoV-2 infection.

### SARS-CoV-2 receptor ACE2

An analysis based on decades-long structural studies of SARS coronavirus revealed that SARS-CoV-2 may utilize angiotensin-converting enzyme-2 (ACE2) as its host receptor, consistent with its capacity for human cell infection and human-to-human transmission [112]. ACE2 is the SARS-CoV-2 docking receptor, and transmembrane protease serine-2 (TMPRSS2) is the main enzyme for proteolysis of the viral spike protein [113]. Both the receptor and enzyme are abundantly expressed in multiple cell types within various organs such as oral and nasal mucosa, olfactory region, lung, heart, esophagus, kidney, bladder, ileum, vasculature, and brain, which is consistent with the multiorgan impairments and tissue damage in COVID-19 patients [22, 114–117]. Meta-analysis of single-cell RNA-seq datasets for putative SARS-CoV-2 targets revealed that ACE2 acts in concert with TMPRSS2 or cathepsin L to promote its cellular entry in specific cell subsets across tissues [118, 119]. They also identified ACE2 as a human species-specific interferon (IFN)-stimulated gene. A recent study resolved the crystal structure of SARS-CoV-2 and demonstrated that this virus interacts with human ACE2 via the C-terminal domain of the spike protein and it displays a stronger affinity for receptor binding than SARS-CoV does [120].

It has been reported that glial cells and neurons express ACE2 receptors, and SARS-CoV can invade the brain primarily via the olfactory bulb and cause neuronal death in mice [121–123]. Autopsy examinations of the patients with acute SARS-CoV illness have also demonstrated the presence of the virus in the brain or CSF, as indicated by electron microscopy, immunohistochemistry, and real-time RT-PCR testing [121, 124]. In addition, immunohistochemistry showed heterogeneous expression of the SARS-CoV-2 receptor ACE2 in the human upper and lower respiratory tract, which may be related to the susceptibility and/or severe disease development to COVID-19 [125]. Besides in the lung, ACE2 and TMPRSS2 are also highly expressed in the human peripheral and CNS including enteric neurons of the small and large intestine, choroid plexus epithelial cells, excitatory and inhibitory neurons, astrocytes, and oligodendrocytes [101, 126, 127].

### Cellular transportation via olfactory mucosa and its nervous projections

SARS-CoV-2 could infect the CNS via retrograde or anterograde axonal transport, neuron-to-neuron or neuron-to-nonneuronal propagation, or through actions on the olfactory, vagus, and trigeminal nerves. Bulk and single-cell RNA sequencing in both humans and mouse revealed that ACE2 and coronavirus cell entry-related genes are expressed in respiratory epithelium and olfactory epithelium, but not in olfactory sensory neurons or olfactory bulb neurons, indicating that SARS-CoV-2 may infect nonneuronal cell types in the olfactory bulb and thus lead to anosmia in COVID-19 patients [128].

Similar to other coronaviruses, SARS-CoV-2 can attack the olfactory bulb or peripheral nerves such as the trigeminal nerve, which connect the brainstem with different organs of the respiratory tract and then affect the CNS during or after infection [129]. Previous studies in mice showed that the neurotropic influenza virus could invade the CNS from the respiratory mucosa and the vagus nerve directly [130]. SARS-CoV-2 was directly injected into deer mice, and immunohistochemistry examinations showed the presence of SARS-CoV-2 in trigeminal ganglionic neurons, neurons, and microglia of the afferent nerves, and the glomerular layer of the olfactory bulb, indicating that this virus may enter into the brain via the gustatory-olfactory-trigeminal pathway [131]. SARS-CoV-2 was also shown to invade the CNS in rhesus monkeys primarily via the olfactory bulb, subsequently spreading to multiple brain regions, including the hippocampus, thalamus, and medulla oblongata, and causing neuroinflammation and local pathological changes [132]. Autopsies from COVID-19 patients detected the presence of SARS-CoV-2 RNA and protein in olfactory mucosal, cerebellum, and cortical neurons [106, 133]. Moreover, analysis of autopsy material from 33 deceased COVID-19 patients revealed colocalization of SARS-CoV spike protein with various neuronal markers in olfactory mucosa, indicating that SARS-CoV-2 can invade the CNS via crossing the olfactory mucosal–neural interface [133]. A postmortem case series also revealed the presence of SARS-CoV-2 viral proteins in cranial nerves originating from the lower brainstem and in isolated cells of the medulla oblongata [134]. In addition, the enteric nervous system and its vagal afferents to the CNS could be alternative targets for SARS-CoV-2 neuroinvasion, considering the large amount of ACE2 receptor expression and the prominent gastrointestinal symptoms in COVID-19 patients [135].

### Hematogenous dissemination via blood–brain and blood–CSF barriers

The coronavirus could diffuse into the CNS through the dissemination of peripherally infected immune cells including monocytes, neutrophils, and T cells, or via binding to the ACE2 receptors in endothelial cells of the BBB or the blood–CSF barrier in the choroid plexus. Single-nucleus transcriptomes from postmortem choroid plexus samples from COVID-19 patients revealed the dysfunction of choroid plexus barrier cells and peripheral T cell infiltration [136]. Emerging evidence indicates that SARS-CoV-2 could infect CNS cells, especially the brain microvascular endothelial cells, thus damaging the BBB and blood–CSF integrity and causing widespread neuroinflammation, which ultimately contributes to the neuropsychiatric complications in COVID-19 patients [101, 137]. In addition, ACE2 is a vasoconstrictor and exerts pro-inflammatory function [138], and SARS-CoV-2 may act on ACE2 in the brain, leading to arterial wall rupture and intracranial bleeding in COVID-19 patients [63]. The systemic inflammation induced by SARS-CoV-2 infection is also likely to increase the barrier permeability, thus promoting peripheral infected granulocytes, systemically released inflammatory cytokines, and possibly the coronavirus itself invade into the CNS.

### Hypoxia, hyperinflammation, and neuroimmune deficits

Growing evidence indicates that SARS-CoV-2 can induce hypoxic conditions caused by lung injury and modulate innate and adaptive immune responses in the host, thus further promoting SARS-CoV-2 neuroinvasion from the periphery to the brain [139]. A case series of radiographic and clinical neurologic presentations showed that hypoxemia secondary to COVID-19-related acute respiratory distress syndrome plays a critical role in hypoxia neuronal injury and neurocognitive impairment [140]. Dysregulation of the brain–lung–heart interactions could also cause hypoxic–ischemic brain damage, hyperinflammation, and procoagulative states, which would further contribute to the neurological manifestations of SARS-CoV-2 infection [141]. In addition to fighting against the coronavirus infection, the immune system is activated and CD4^+^ T cells produce granulocyte–macrophage colony-stimulating factor, which further promotes the overproduction and release of leukocytes and pro-inflammatory cytokines, especially interleukin-6 (IL-6), causing overactivation of the complement system and coagulation cascades, and ultimately contributing to a vicious cycle of the cytokine storm and adverse clinical outcomes [142–144]. Astrocytes and microglia, the resident immune cells in the brain, also play a critical role in SARS-CoV-2 infection, neuroinflammation, and CNS injury [145]. After a neurotropic coronavirus infection, microglia is required for debris clearance and the initiation of remyelination [146], which may be correlated with spinal demyelinating lesions in COVID-19 patients.

COVID-19-related neuropsychiatric symptoms such as encephalopathy, stroke, depression, and post-traumatic stress disorder are associated with cytokine release syndrome, BBB dysfunction (as indicated by hyper-albumin-orrachia and increased astroglial protein S100B levels), neuroinflammatory, neurochemical changes, and immune-mediated mechanisms [147, 148]. Immune analysis on a cohort of 50 COVID-19 patients with various disease severities revealed impaired type I IFN activity, characterized by no IFN-β and low IFN-α production. These low IFN levels were associated with a persistent viral load in the blood and an exacerbated inflammatory response (overproduction and release of tumor necrosis factor-α [TNF-α] and IL-6), which may be a hallmark of severe COVID-19 [149]. Moreover, IL-6 and TNF-α levels have been shown to predict disease severity, clinical progression, and death in hospitalized COVID-19 patients [150, 151]. A case report demonstrated a marked increase of several inflammatory cytokines and chemokines in the CSF of a COVID-19 patient [152]. Single-cell sequencing analysis of CSF immune cells from COVID-19 patients with neurological manifestations revealed expansion of dedifferentiated monocytes, exhausted CD4^+^ T cells, and reduced IFN response [153]. Autopsies of COVID-19 patients revealed astrocytosis, axonal damage, BBB leakage, and alterations of CD8^+^ T cell–microglia crosstalk in the CNS [154]. These findings indicate that CNS inflammation and immune-mediated mechanisms may contribute to COVID-19-related long-lasting neurological sequelae.

Hyperinflammatory syndrome in COVID-19 manifested as fever, macrophage activation, hematological dysfunction, hepatic injury, coagulopathy, and cytokinemia, is closely associated with worse respiratory symptoms and in-hospital mortality [155]. Severe COVID-19 patients also mostly exerted viral infection-induced hyperinflammatory state, mainly characterized by sustained increases in TNF-α, IL-6, and IL-1 levels, and disrupted monocyte and dendritic cell phenotypes, which could ultimately contribute to poor prognosis and mortality [151, 156]. A meta-analysis revealed that increased neutrophil-to-lymphocyte ratios and decreased lymphocyte-to-CRP ratios, which are two markers of systemic inflammation, were found in severe COVID-19 patients and may indicate a poor prognosis [157]. Synergism of TNF-α and IFN-γ triggered lethal cytokine shock syndromes and treatment with their neutralizing antibodies prevented SARS-CoV-2 infection-induced mortality in mice [158]. Cross-reactive antibodies to SARS-CoV-2 may also contribute to COVID-19-related disease pathology and the persistence of neurological symptoms even in recovered patients [159]. Sex differences in immune responses also appear to make males more susceptible to SARS-CoV-2 infection, and worse clinical outcomes that are associated with poor T cell-mediated immunity in male COVID-19 patients [160, 161].

Central and peripheral immunological responses to SARS-CoV-2 infection are distinct. Immune analysis of mononuclear cells from COVID-19 patients with neurological symptoms revealed unique B cell responses that markedly differed between the CSF and peripheral blood [162]. Furthermore, a mouse model of SARS-CoV-2 infection found that antibody responses can occur separately in the brain without being evident in the systemic circulation. Specifically, a case report showed COVID-19-related meningitis without respiratory manifestations and negative oropharyngeal/nasopharyngeal RT-PCRs, but the CSF RT-PCR assay was positive [163]. A case report also showed a patient with serious neurological damage and mental abnormalities without respiratory symptoms, but with strong IgM and IgG antibodies against SARS-CoV-2 in the CSF. This patient also had negative RT-PCR results in both nasopharyngeal swabs and CSF [164].

COVID-19 appears to affect children more mildly than adults, which may be due to the reduced ACE2 level in children’s respiratory tract, their protective T cell and T-helper 2 (Th2) immunity, and their decreased inflammatory responses [165]. However, a multisystem inflammatory syndrome in children (MIS-C) is fatal and worrisome and has been increasingly reported and noticed [166]. A case report of a 4-year-old COVID-19 child presenting with MIS-C and prominent neurologic symptoms, and described how a cytokine storm and decreased BDNF level may contribute to his neurocognitive dysfunction [167]. The increased concentrations of CRP and IL-6 are strongly associated with severe outcomes in COVID patients with MIS-C [168]. A population-based longitudinal study revealed that higher levels of the systemic inflammatory marker IL-6 at age 9 years were more likely to develop depression and psychosis at age 18 years [169]. Mechanically, maternal inflammation affects multiple steps of cortical GABAergic interneuron development, thus contributing to the cognitive impairment of the affected offspring [170]. Furthermore, early-life mental health has been associated with decreased levels of fibrinogen and CRP, two indicators of inflammation and cardiovascular disease, and increased risk for all-cause mortality in later life [171].

**Fig. 2:**
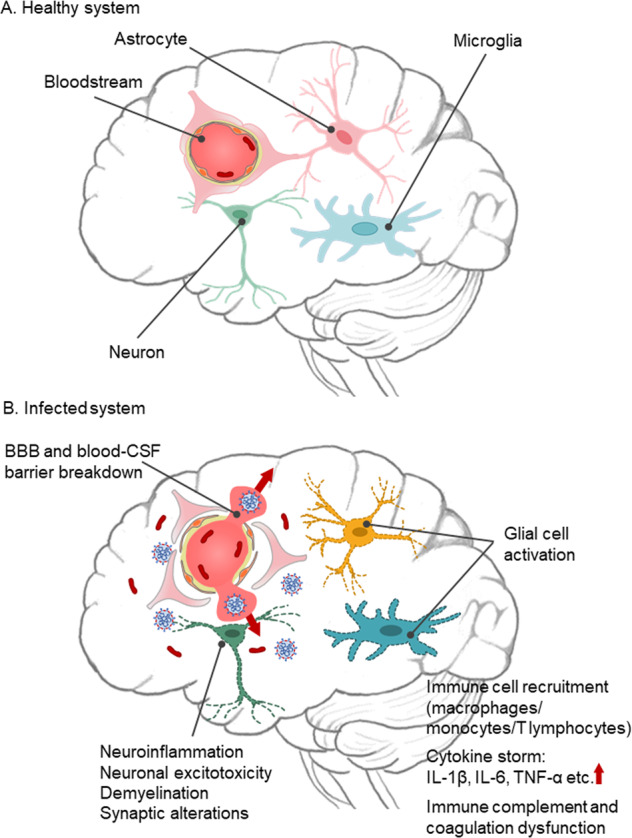
Schematic illustration of proposed consequences of SARS-CoV-2 neuroinvasion. **A** In healthy systems, the blood-rain barrier (BBB) prevents most of the macromolecules and neurotoxins in the bloodstream from entering the brain tissues, which is achieved through the components of the neurovascular unit (e.g., endothelial cells, astrocytes). **B** Growing evidence suggests that SARS-CoV-2 may invade brain tissue. The virus may enter the bloodstream through multiple pathways and infect the neurovascular cells, causing the breakdown of the BBB and blood–cerebrospinal fluid (CSF) barrier. Their breakdown will further trigger the inflammatory responses, including the recruitment of macrophage and T lymphocytes, activation of astrocytes and microglia, cause a vicious cycle of the cytokine storm (production and release of IL-1β, IL-6, TNF-α, etc.), and disrupt immune complement and coagulation cascades. The infection may also cause immune-mediated demyelination, neuronal excitotoxicity, and dysfunction of synaptic plasticity, which ultimately contributes to worsened neurological and neuropsychiatric symptoms in COVID-19 patients.

Overall, the SARS-CoV-2 may directly invade the CNS via cellular transportation and hematogenous dissemination by acting on ACE2 receptors in endothelial cells of BBB or the blood–CSF barrier, and indirectly damage the brain via hypoxia, hyperinflammation, and neuroimmune system. This leads to the recruitment and activation of immune cells including resident microglia and astrocytes, and peripheral leukocytes and macrophages, which increases the production and release of inflammatory cytokines and chemokines, further induces the breakdown of BBB and blood–CSF barriers, and causes neuroinflammation, demyelination, neuronal excitotoxicity, and synaptic plasticity deficits in the brain, forming a vicious cycle of the cytokine storm and neuronal injury (Fig. 2). The dysfunction of the peripheral and CNS could ultimately contribute to the neurologic and neuropsychiatric manifestations in COVID-19 patients.

## Strategies for treatment of SARS-CoV-2-related neuropsychiatric complications

As the neurologic and neuropsychiatric manifestations are commonly reported in COVID-19 patients, especially in severe conditions, there is an urgent need to develop effective strategies and therapeutic frameworks to prevent and reduce them in the clinic (Table 3).

**Table 3:** Treatment of COVID-19-associated neuropsychiatric complications.

Study	Region	Participants	Sample size	Study design	Symptoms	Interventions (*n* patients)	Clinical outcomes	Main findings
ACEIs/ARBs								
De Spiegeleer et al., 2020 [176]	Belgium	COVID-19-diagnosed residents	154	Retrospective cohort	Not specified	ACEIs/ARBs (30), statin (31)	ACEIs/ARBs (6 serious, 24 nonserious), statin (6 serious, 25 nonserious)	Statin treatment associated with beneficial effects on COVID-19-related clinical symptoms, statin treatment combined with ACEI or ARB associated with less severe clinical outcomes
Luzzi et al., 2020 [179]	Italy	COVID-19 patients affected by stroke	6	Case series	Not specified	ACEIs/ARBs	Recovery	Unclear whether and how pharmacomodulation of the renin–angiotensin system affected the clinical course of COVID-19 patients who were affected by stroke
Matsuzawa et al., 2020 [175]	Japan	COVID-19 patients (39 with hypertension)	151	Retrospective cohort	Mental confusion related to pneumonia (14)	ACEIs/ARBs (21), non-ACEIs/ARBs (18)	In-hospital death (14), mechanical ventilation (14), oxygen therapy (58)	ACEIs/ARBs could be beneficial for the prevention of confusion in COVID-19 patients with hypertension
Meng et al., 2020 [173]	China	COVID-19 patients with hypertension	42	Retrospective cohort	Not specified	ACEIs/ARBs (17), non-ACEIs/ARBs (25)	ACEIs/ARBs (4 severe, 0 died), non-ACEIs/ARBs (12 severe, 1 died)	Renin–angiotensin system inhibitors improve clinical outcomes of COVID-19 patients with hypertension
Zhang et al., 2020 [174]	China	Patients with hypertension and COVID-19	1128	Retrospective cohort	Non-ACEI/ARB group had a higher prevalence of fever, dyspnea, and bilateral lung lesion	ACEIs/ARBs (188), non-ACEIs/ARBs (940)	99 deaths out of 1128 patients, risk of 28-day all-cause mortality was significantly lower in ACEI/ARB group	Among hospitalized patients with COVID-19 and coexisting hypertension, inpatient use of ACEI/ARB associated with a lower risk of all-cause mortality compared with ACEI/ARB nonusers
Intravenous immunoglobulin								
Khaja et al., 2020 [196]	USA	COVID-19 with Guillain–Barré syndrome	1	Case report	Bilateral facial weakness, unable to raise eyebrows, unable to close eyes, unable to smile, loss of taste sensation	Intravenous immunoglobulin	Discharged on day 12	Infection with SARS-CoV-2 resulted in acute neurological involvement, leading to loss of taste, Guillain–Barré syndrome, and facial weakness
Manganotti et al., 2021 [195]	Italy	COVID-19 patients with drug-resistant status epilepticus	2	Case series	Convulsive status epilepticus, severe respiratory failure symptoms	Intravenous immunoglobulin (0.4 g/kg)	Discharged from hospital	Support the use of Intravenous immunoglobulin therapy in new-onset refractory status epilepticus COVID-19 patients with suspected autoimmune encephalitis to prevent negative outcomes
Muccioli et al., 2021 [194]	Italy	COVID-19 patients	5	Case series	Encephalopathy, confusion, delirium, agitation, akinetic mutism, apraxia, and pyramidal, extrapyramidal, and frontal release signs	Intravenous immunoglobulin (0.4 g/kg)	Complete electroclinical recovery	Intravenous immunoglobulin may be a safe and effective treatment for COVID-19-associated encephalopathy
Tocilizumab								
Dastan et al., 2020 [189]	Iran	Severe or critical SARS-CoV-2 patients	42	Noncontrolled trial	Receiving oxygen via face mask (31), receiving oxygen via nasal cannula (11)	Tocilizumab (20 severe, 22 critical)	Severe (19 survivors, 1 non-survivor), critical (16 survivors, 6 non-survivors)	Tocilizumab may be promising for patients with severe or critical SARS-CoV-2 infection if promptly initiated during the severe stage
Muccioli et al., 2020 [192]	Italy	COVID-19-related encephalopathy	1	Case report	Expressive aphasia, inattentiveness, agitation, marked confusion	Tocilizumab	Discharged asymptomatic	Neuropsychiatric symptoms resolved following treatment with tocilizumab, future studies should further investigate the role of tocilizumab in treating COVID-19-related encephalopathy
Talluri et al., 2021 [193]	USA	COVID-19 patients with encephalopathy	1	Case report	Fever, cough, exertional dyspnea, hypoactive delirium	Tocilizumab	Died on day 27	Clinicians should consider posterior reversible encephalopathy syndrome in differential diagnoses when managing COVID-19 patients with encephalopathy, especially those who receive immunotherapy (e.g., tocilizumab)

*ACEIs/ARBs* angiotensin-converting enzyme inhibitors/angiotensin receptor blockers.

### ACEIs/ARBs

Previous studies showed that ACE2 expression level was correlated with susceptibility to SARS-CoV infection and the development of neuropsychiatric symptoms [172]. Angiotensin-converting enzyme inhibitors (ACEIs)/angiotensin receptor blockers (ARBs) are commonly used in the treatment of cardiovascular diseases such as hypertension and coronary heart disease. Comorbidity with hypertension has mostly been reported among patients who died of COVID-19. Many of these patients used renin–angiotensin system inhibitors, such as ACEIs and ARBs, which increase ACE2 receptor expression at the cell membrane in experimental animals, and theoretically this larger number of ACE2 receptors enhance viral entry and lead to a higher risk of SARS-CoV-2 infection [116, 138]. However, little current human evidence supports this assertion. Recent clinical evidence indicates that the use of these drugs improves clinical outcomes by reducing the occurrence of new-onset or worsening mental confusion (e.g., mild disorientation or hallucinations) and decreases the risk of all-cause mortality in COVID-19 patients with hypertension [173–175]. A retrospective multicenter cohort study reported that statin treatment in combination with an ACEI or ARB had beneficial, albeit nonsignificant, effects on serious clinical outcomes of COVID-19 in older adults who lived in nursing homes [176]. Society and practice guidelines recommend continued therapy for patients who have previously prescribed these drugs for another indication [177]. ACEIs/ARBs should not be discontinued for COVID-9 infected patients as their protective role in heart injuries and lung damage caused by the infection. However, it is not recommended to initiate ACEIs/ARBs because there has been no definitive evidence that they benefit COVID- 19 patients’ survival [138, 178]. Randomized clinical trials are needed to further clarify the safety and efficacy of ACEIs/ARBs in COVID-19 patients with neuropsychiatric complications such as stroke [179].

### Anti-inflammatory drugs and interventions

Early adaptive immune responses, such as the recruitment of multiple immune cells and the concomitant production of immunoglobulin antibodies, may predict better clinical outcomes in COVID-19 patients [180]. During aging, immune system function declines, and adaptive immunity fails to develop, which can significantly weaken the defense response to SARS-CoV-2 infection in the elderly and worsen clinical outcomes. Because of the risk for a cytokine storm syndrome in a subgroup of patients with severe COVID-19, immunosuppression is recommended for COVID-19 treatment [156]. Immunosuppressive agents are also widely used to combat several disorders of both the central and peripheral nervous systems such as multiple sclerosis. Corticosteroids were commonly used during the outbreaks of SARS-CoV and MERS-CoV, especially for patients with critical conditions [181, 182]. Corticosteroids inhibit systemic inflammation, but also suppress immune activity and prevent viral clearance. Some clinical evidence suggests that corticosteroid treatment had no beneficial effects on SARS-CoV-2-induced lung injury or mortality [183–185]. Conversely, a meta-analysis revealed that low-dose corticosteroid therapy appeared to reduce all-cause mortality in critically ill patients with COVID-19 [186, 187]. Corticosteroids were also reported to exert potential beneficial effects on COVID-19-associated neurological disorders, including encephalitis and acute disseminated encephalomyelitis [188].

IL-6 is prominent in the cytokine storm that underlies COVID-19-related acute respiratory distress syndrome and brain injury. Increasing evidence supports the safety and efficiency of the IL-6 receptor blocker tocilizumab for treating patients with severe or critical COVID-19, especially those with cytokine release syndrome [189–191]. Case reports described that tocilizumab exerted potential beneficial effects on COVID-19-related neuropsychiatric symptoms, including encephalopathy presenting with aphasia and posterior reversible encephalopathy syndrome [192, 193]. Case reports and case series showed that immunotherapy with intravenous immunoglobulin was safe and effective for the treatment of COVID-19-associated neurological disorders, including encephalopathy, GBS, and new-onset refractory status epilepticus [194–196]. Janus kinase inhibitor baricitinib has been shown to reduce macrophage and neutrophil recruitment and suppress inflammation in a rhesus macaque model of SARS-CoV-2 infection [197]. Baricitinib treatment could affect both inflammation and cellular viral endocytosis and entry into cells, thus it could be a potential treatment for COVID-19 [198]. Thiamine, a vitamin and dietary supplement, was recently reported to attenuate the Th17 cell-mediated IL-17 pro-inflammatory cytokine storm related to COVID-19, and is a promising repurposed drug for treating neurological symptoms in COVID-19 patients [199]. Meta-analysis of randomized clinical trials showed that psychosocial interventions are associated with enhanced immune system function for at least 6 months following treatment cessation, as indexed by decreases in levels of pro-inflammatory cytokines or markers (e.g., IL-6, CRP), and by increases in immune cell counts (e.g., CD56, CD4) [200]. Psychosocial interventions, including cognitive behavioral therapy, psychological first aid, and a community-based psychosocial arts program, were reported to have beneficial effects on psychological and psychiatric complications in COVID-19 patients [201].

### Other approaches

Large-scale compound repurposing of known antiviral drugs identified several small molecules, such as the PIKfyve kinase inhibitor and the cysteine protease inhibitors, as candidate therapeutic drugs for COVID-19 [202]. Antimalarials such as chloroquine and hydroxychloroquine were found to counter neuroinflammation and have promising beneficial effects on COVID-19-related neurological/neurovascular complications [203], although randomized clinical trials showed that hydroxychloroquine had no efficacy to prevent transmission of SARS-CoV-2 among hospital-based healthcare workers [204], and was associated with increased mortality in COVID-19 patients [205]. In addition, potential neuropsychiatric side effects of chloroquine and hydroxychloroquine suggest avoiding them as COVID-19 treatments, and if these agents are used, evaluation of patients’ psychiatric presentations such as psychosis, mood disorders, and suicide risk should be implemented [206–208]. A vaccine BNT162b1 targeting the receptor-binding domain of the SARS-CoV-2 spike protein elicited robust T cell and strong human antibody responses, indicating promising therapeutic and prevention potential against COVID-19 [209]. A pilot randomized controlled study intends to evaluate the effects of low-intensity transcranial direct current stimulation on dyspnea relief in COVID-19 patients requiring mechanical ventilation in ICU [210]. In addition, intranasal delivery is proposed in the management of neurological and neuropsychiatric manifestations of COVID-19 as it could overcome the BBB impediment and enter the CNS, with minimal adverse effects [211].

## Conclusions and perspectives

Neurological and neuropsychiatric symptoms are common in COVID-19 patients. Emerging neuroimaging and neurochemical evidence indicate neuroimmune dysfunction and brain injury in severe COVID-19 patients, especially those with neuropsychiatric manifestations. These neuropsychiatric complications are recognized as critical contributors to morbidity and mortality during the COVID-19 pandemic and have become major public health challenges. SARS-CoV-2 infection can directly invade the CNS through the blood circulation and neuronal pathways, and indirectly affect the innate and adaptive immune system and cause neuroinflammation. Both of these disruptions to immune functioning and neuroinflammation ultimately lead to brain lesions and accelerate the progression and worsening of the clinical outcomes of neuropsychiatric disorders.

Immun-suppressive therapies, vaccines targeting the coronaviruses’ spike protein, and pharmacological agents that improve endothelial integrity, reduce hypercoagulation status or targeting the host’s ACE2 receptor, to which the virus binds, could prevent neuropsychiatric complications such as stroke and benefit an individual’s mental health such as poststroke depression in COVID-19 patients (Fig. 1). However, to date, no pharmacologic guidelines or standard protocols exist for the management of neuropsychiatric symptoms in COVID-19 patients. Integrated multilayered and multidisciplinary research including epidemiology, pharmacology, imaging, translational experimental studies, and randomized clinical trials are urgently needed to develop safe and effective interventions to reduce the short- and long-term neuropsychiatric complications in hospitalized or recovered patients with COVID-19.

Some neurological and neuropsychiatric manifestations may be due to systemic involvement rather than direct CNS infection. Thus, it should be made with caution when lacking solid evidence for neuroinvasion in COVID-19 patients with neurological symptoms such as the absence of viral detection in CSF samples or abnormalities in brain imaging. Further experimental animal studies, prospective or retrospective observational studies, randomized clinical trials, autopsies, and CSF analysis are urgently needed to elaborate the direct or indirect effects of SARS-CoV-2 infection on different cell types in the CNS, spinal cord, and peripheral nervous system, clarify the neuroinvasive pathways of this devastating virus and confirm the causative roles of inflammation or immune system dysfunction, hypoxic–ischemic brain injury, and hypercoagulability in disease pathology. These clinical–epidemiological laboratory and biological mechanism investigations will ultimately guide the clinic to prioritize and individualize therapeutic protocols based on the disease severity, neuropsychiatric presentations, and predominant organ involvement. Awareness and management of these neuropsychiatric complications are critical to improve the prognosis and reduce mortalities of critically ill COVID-19 patients.

### Search strategy and selection criteria

References for this review were identified by searching PubMed for articles on COVID-19 from database inception to August 2021, without language restrictions. The following search terms were used: “COVID-19”, “SARS-CoV-2”, “neurological”, “psychiatric”, “neuropsychiatric”, “neurotropism”, “neuroinvasion”, “nervous system”, “treatment”, and “intervention”. Additional articles were identified by searching the references of relevant articles. The final list of included articles was generated based on relevance and originality with regard to the topics covered in this review.
